# Supplementary material for: Functional improvement is a better predictor of steady work than medical improvement for individuals with mental health conditions
Source: PLOS Ment Health. 2025 Aug 5;2(8):e0000384. doi: 10.1371/journal.pmen.0000384 (PMC12798178; doi:10.1371/journal.pmen.0000384)
Supplement: S1 Text — (PDF) [file pmen.0000384.s001.pdf]

S1 Text to Functional improvement is a better predictor of steady work than medical improvement for individuals with mental health conditions

Joshua C. Chang Julia Porcino Elizabeth Marfeo Larry Tang
Howard Goldman Elizabeth Rasch

July 14, 2025

S1 Supplemental Methods

We model the probability of steady work for participant n in year j using the likelihood

$$y_{n,j} \sim \text{Bern}(\sigma(\mu_n)) \qquad (1)\ \mu_{n,j} = \alpha_n + \mathbf{x}_{n,j}\beta \qquad (2)$$

where σ is the sigmoid function, and $\mathbf{x}_{n,j} \in \mathbb{R}^p$ is the associated vector of predictors of dimension p. In this formulation, the person-specific parameters α_n are random intercept terms.

S1.1 Missingness

We used marginalization in order to resolve missingness in the dataset. We first determined whether each predictor variable is a count, or otherwise real-valued. We then fit predictive models to each predictor variable, simultaneously for integer count-based predictors

$$x_{n,j,y} \sim \text{Poisson}(r_{n,j,y}) \qquad r_{n,j} = \gamma_{n,j} + \sum_{k=j} \eta_k x_{n,k,y},$$

and otherwise real-valued predictors

$$x_{n,j,y} \sim \text{Normal}(\sigma(r_{m,j,y}),\sigma_j) \qquad r_{n,j,y} = \gamma n,j + \sum_{k=j} \eta_k x_{n,k,y},$$

for each variable j, year y, and person n. In essence we model each unknown value for a given year as a generalized linear regression on the other values for that year and a person-specific random effect that is pooled across years.

In sampling the full model's posterior distribution and computing the statistics for β and α_n, we marginalize over all of the model parameters for the missing observations.

S1.2 Bayesian model priors

We regularize the model by using a combination of weakly informative and sparsity promoting priors,

$$\alpha_n \sim \text{normal}(0,10)$$
$$\beta \sim \text{Finnish-Horseshoe}(n), \qquad (3)$$

where the Finnish horseshoe prior [1, 2] promotes sparsity-based regularization on β.

S2 Supplemental Results

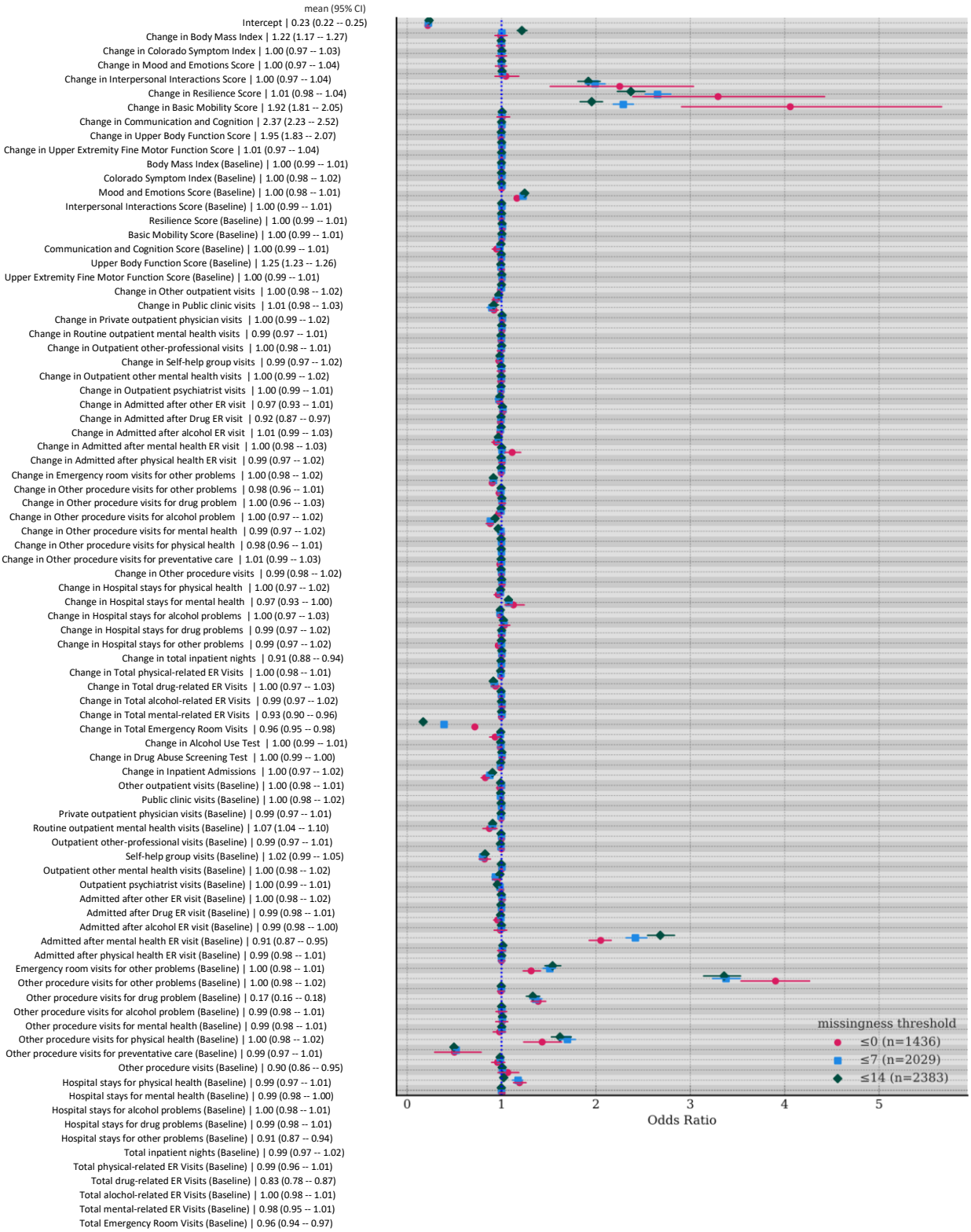

Alcohol Use Disorders Identification Test (Baseline) | 1.00 (0.98 -- 1.02)
Drug Abuse Screening Test score (Baseline) | 0.99 (0.98 -- 1.01)
Inpatient Hospital Admissions (Baseline) | 0.99 (0.97 -- 1.01)
Has Wheelchair | 1.00 (0.97 -- 1.03)
Has Community Mobility Drive Score | 2.68 (2.55 -- 2.83)
Has Community Mobility Ride Score | 1.01 (0.99 -- 1.05)
Age 35+ (Baseline) | 1.00 (0.98 -- 1.03)
Worked in Past 2 Years (Baseline) | 1.54 (1.46 -- 1.63)
Working Status (Baseline) | 3.36 (3.14 -- 3.53)
Ever Worked (Baseline) | 1.00 (0.97 -- 1.02)
Study arm assigment to Treatment group | 1.33 (1.26 -- 1.40)
Multiple Residences past 30 days (Baseline) | 1.00 (0.97 -- 1.03)
Lived in homeless shelter past 30 days (Baseline) | 1.01 (0.98 -- 1.04)
Living Alone past 30 days (Baseline) | 1.00 (0.97 -- 1.03)
Bachelor's Degree | 1.62 (1.53 -- 1.74)
Other Race | 0.50 (0.47 -- 0.53)
Black | 0.98 (0.96 -- 1.01)
Hispanic | 1.01 (0.98 -- 1.03)
Always has access to a car (Baseline) | 1.02 (0.98 -- 1.06)
Healthcare Coverage (Baseline) | 1.00 (0.97 -- 1.02)

Figure A: Comparing models based on missingness thresholds, finding that the models were largely consistent in terms of findings.

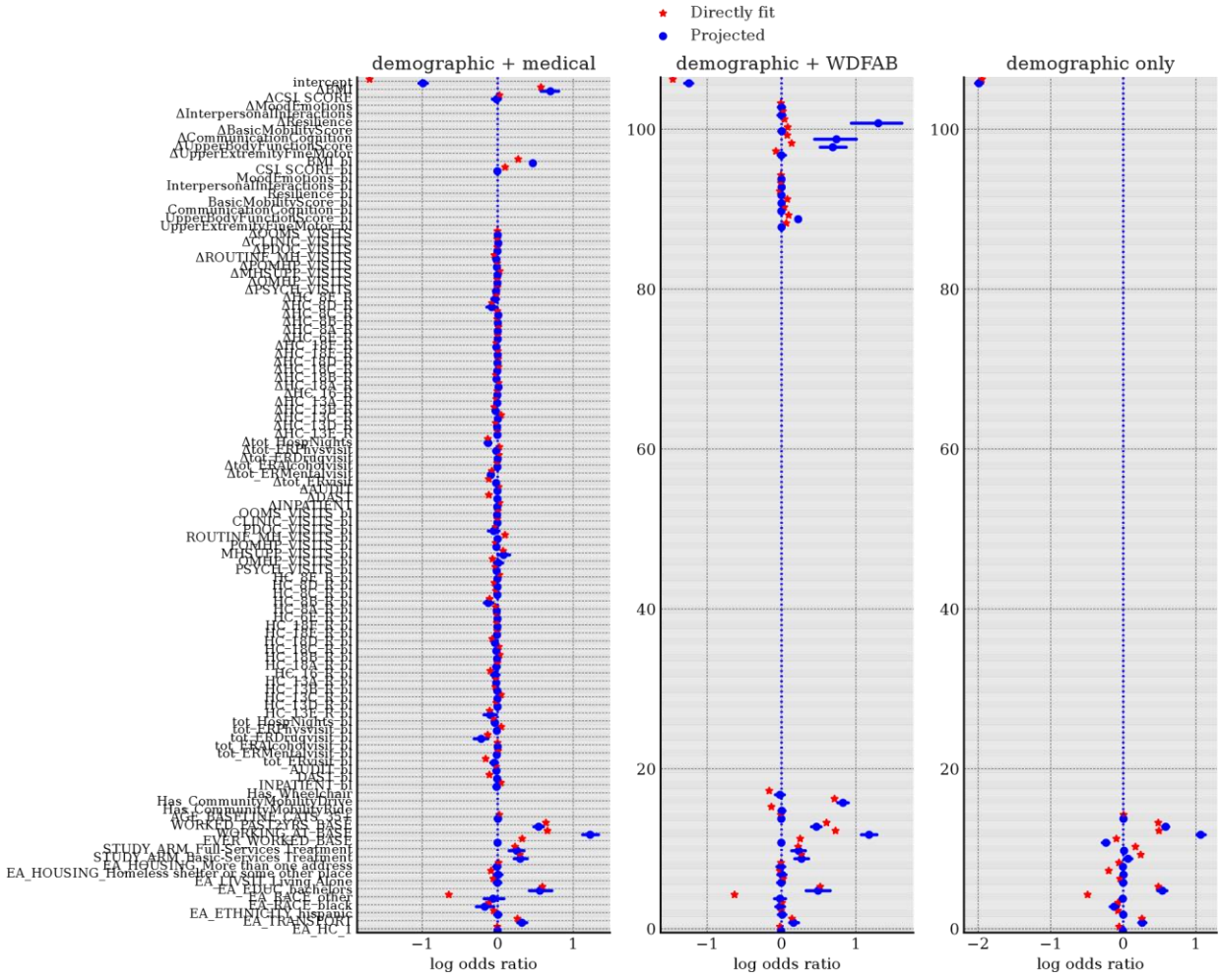

Figure B: Regression coefficients for submodels fitted either directly or through a posterior predictive projection method [3–7]

S3 Source code

The following code describes the model

```

from collections import defaultdict
from bayesianquilts import BayesianModel
import tensorflow as tf
from tensorflow_probability.python import distributions as tfd
from tensorflow_probability.python import bijectors as tfb
from bayesianquilts.vi.advi import build_surrogate_posterior
from bayesianquilts.util import batched_minimize

import numpy as np
import pandas as pd
from sklearn import metrics
from psisloo import psisloo
from tqdm import tqdm
import sys

from nppsis import psisloo, psislw

# mu0 is person/variable specific
# mu00 is variable specific


class ImputedBernModelRescaled(BayesianModel):
    def __init__(
        self,
        n_people,
        gaussian_yearly_vars,
        poisson_yearly_vars,
        binary_yearly_vars,
        baseline_vars,
        outcome_var=None,
        outcome_tau=1.0,
        beta_outcome_scale=2.0,
        init=None,
        means=None,
        stds=None,
        diff_means=None,
        diff_stds=None,
        dtype=tf.float64,
    ):
        if outcome_var is None:
            outcome_var = "STEADY_WORKER"
        self.outcome_var = outcome_var
        self.outcome_tau = outcome_tau
        self.dtype = dtype
        self.strategy = None
        self.gaussian_yearly_vars = gaussian_yearly_vars
        self.diff_means = diff_means if diff_means is not None else defaultdict(float)
        self.diff_stds = diff_stds if diff_stds is not None else defaultdict(lambda: 1)
        self.means = means if means is not None else defaultdict(float)
        self.stds = stds if stds is not None else defaultdict(lambda: 1)
        self.gaussian_yearly_vars_loc = tf.cast(
            [self.means.get(c, 0) for c in gaussian_yearly_vars], self.dtype
        )
        self.gaussian_yearly_vars_scale = tf.cast(
            [self.stds.get(c, 1) for c in gaussian_yearly_vars], self.dtype
        )
        self.gaussian_yearly_vars_diff_loc = tf.cast(
            [self.diff_means.get(c, 0) for c in gaussian_yearly_vars], self.dtype
        )
        self.gaussian_yearly_vars_diff_scale = tf.cast(
            [self.diff_stds.get(c, 1) for c in gaussian_yearly_vars], self.dtype
        )
        self.binary_yearly_vars = binary_yearly_vars
        self.poisson_yearly_vars = poisson_yearly_vars
        self.poisson_yearly_vars_loc = tf.cast(
            [self.means.get(c, 0) for c in poisson_yearly_vars], self.dtype
        )
        self.poisson_yearly_vars_scale = tf.cast(
            [self.stds.get(c, 1) for c in poisson_yearly_vars], self.dtype
        )
```

```
        self.poisson_yearly_vars_diff_loc = tf.cast(
            [self.diff_means.get(c, 0) for c in poisson_yearly_vars], self.dtype
        )
        self.poisson_yearly_vars_diff_scale = tf.cast(
            [self.diff_stds.get(c, 1) for c in poisson_yearly_vars], self.dtype
        )
        self.baseline_vars = baseline_vars
        self.beta_outcome_scale = beta_outcome_scale
        self.n_people = n_people
        self.init = init

        self.create_distributions(init=init)

    def create_distributions(self, init=None):
        self.prior_distribution = tfd.JointDistributionNamed(
            {
                "alpha_outcome": tfd.Independent(
                    tfd.Normal(
                        loc=tf.zeros([1], self.dtype),
                        scale=10 * tf.ones([1], self.dtype),
                    ),
                    reinterpreted_batch_ndims=1,
                ),
                "mu0_gaussian": tfd.Independent(
                    tfd.Normal(
                        loc=tf.zeros(
                            [self.n_people, len(self.gaussian_yearly_vars), 1],
                            dtype=self.dtype,
                        ),
                        scale=3
                        * tf.ones(
                            [self.n_people, len(self.gaussian_yearly_vars), 1],
                            dtype=self.dtype,
                        ),
                    ),
                    reinterpreted_batch_ndims=3,
                ),
                "mu0_poisson": tfd.Independent(
                    tfd.Normal(
                        loc=tf.zeros(
                            [self.n_people, len(self.poisson_yearly_vars), 1],
```

```
                                    dtype=self.dtype,
                                ),
                                scale=3
                                * tf.ones(
                                    [self.n_people, len(self.poisson_yearly_vars),  1],
                                    dtype=self.dtype,
                                ),
                            ),
                            reinterpreted_batch_ndims=3,
                        ),
                        "mu00_gaussian": tfd.Independent(
                            tfd.Normal(
                                loc=tf.zeros(
                                    [1, len(self.gaussian_yearly_vars), 1], dtype=self.dtype
                                ),
                                scale=6
                                * tf.ones(
                                    [1, len(self.gaussian_yearly_vars), 1], dtype=self.dtype
                                ),
                            ),
                            reinterpreted_batch_ndims=3,
                        ),
                        "mu00_poisson": tfd.Independent(
                            tfd.Normal(
                                loc=tf.zeros(
                                    [1, len(self.poisson_yearly_vars), 1], dtype=self.dtype
                                ),
                                scale=5
                                * tf.ones(
                                    [1, len(self.poisson_yearly_vars), 1], dtype=self.dtype
                                ),
                            ),
                            reinterpreted_batch_ndims=3,
                        ),
                        "sd00_gaussian": tfd.Independent(
                            tfd.HalfNormal(
                                scale=tf.ones(
                                    [1, len(self.gaussian_yearly_vars), 1], dtype=self.dtype
                                ),
                            ),
                            reinterpreted_batch_ndims=3,
                        ),
                        "beta_outcome": tfd.Independent(
                            tfd.Horseshoe(
                                # loc=tf.zeros(
                                #     [
                                #         len(baseline_vars + has_vars)
```

```
                        #                     + len(poisson_yearly_vars) * 2
                        #                     + len(gaussian_yearly_vars) * 2
                        #         ],
                        #           self.dtype,
                        # ),
                        scale=self.beta_outcome_scale
                        / np.sqrt(3 * self.n_people)
                        * tf.ones(
                            [
                                len(self.baseline_vars)
                                + len(self.binary_yearly_vars)
                                + len(self.poisson_yearly_vars) * 2
                                + len(self.gaussian_yearly_vars) * 2
                            ],
                            self.dtype,
                        ),
                    ),
                    reinterpreted_batch_ndims=1,
                ),
                "beta_gaussian": tfd.Independent(
                    tfd.Normal(
                        loc=tf.zeros(
                            [
                                len(self.baseline_vars)
                                + len(self.binary_yearly_vars)
                                + len(self.poisson_yearly_vars),
                                len(self.gaussian_yearly_vars),
                            ],
                            self.dtype,
                        ),
                        scale=self.outcome_tau
                        * tf.ones(
                            [
                                len(self.baseline_vars)
                                + len(self.binary_yearly_vars)
                                + len(self.poisson_yearly_vars),
                                len(self.gaussian_yearly_vars),
                            ],
                            self.dtype,
                        ),
                    ),
                    reinterpreted_batch_ndims=2,
                ),
                "beta_poisson": tfd.Independent(
                    tfd.Normal(
                        loc=tf.zeros(
                            [
                                len(self.baseline_vars)
                                + len(self.binary_yearly_vars)
                                + len(self.gaussian_yearly_vars),
                                len(self.poisson_yearly_vars),
                            ],
                            self.dtype,
                        ),
```

```
                                scale=self.outcome_tau
                                * tf.ones(
                                    [
                                        len(self.baseline_vars)
                                        + len(self.binary_yearly_vars)
                                        + len(self.gaussian_yearly_vars),
                                        len(self.poisson_yearly_vars),
                                    ],
                                    self.dtype,
                                ),
```

```
                            ),
                            reinterpreted_batch_ndims=2,
                        ),
                    }
                )
                bijectors = defaultdict(lambda: tfb.Identity())
                bijectors["sd0_gaussian"] = tfb.Softplus()
                bijectors["sd00_gaussian"] = tfb.Softplus()
                initializers = {
                    "beta_gaussian": tf.zeros(
                        [
                            len(self.baseline_vars)
                            + len(self.binary_yearly_vars)
                            + len(self.poisson_yearly_vars),
                            len(self.gaussian_yearly_vars),
                        ],
                        self.dtype,
                    ),
                    "beta_poisson": tf.zeros(
                        [
                            len(self.baseline_vars)
                            + len(self.binary_yearly_vars)
                            + len(self.gaussian_yearly_vars),
                            len(self.poisson_yearly_vars),
                        ],
                        self.dtype,
                    ),
                    "beta_outcome": tf.zeros(
                        [
                            len(self.baseline_vars)
                            + len(self.binary_yearly_vars)
                            + len(self.poisson_yearly_vars) * 2
                            + len(self.gaussian_yearly_vars) * 2
                        ],
                        self.dtype,
                    ),
                    "mu0_gaussian": tf.zeros(
                        [self.n_people, len(self.gaussian_yearly_vars), 1], dtype=self.dtype
                    ),
                    "mu0_poisson": tf.zeros(
                        [self.n_people, len(self.poisson_yearly_vars), 1], dtype=self.dtype
                    ),
                    "sd00_gaussian": tf.ones(
```

```
                    [1, len(self.gaussian_yearly_vars), 1], dtype=self.dtype
                ),
            }
            self.beta_outcome_labels = (
                self.baseline_vars
                + self.binary_yearly_vars
                        + [f"{c}_bl" for c  in self.poisson_yearly_vars]
                         + [f"{c}" for c in  self.poisson_yearly_vars]
                        + [f"{c}_bl" for c  in self.gaussian_yearly_vars]
                         + [f"{c}" for c in  self.gaussian_yearly_vars]
            )

            if init is not None:

                test = self.prior_distribution.sample()
                to_delete = []
                for k, v in init.items():
                    if np.array_equiv(test[k].shape.as_list(), v.shape.as_list()):
                        print(f"Using prior {k}", flush=True)
                        initializers[k] = v
                    else:
                        to_delete += [k]
                for k in to_delete:
                    del self.init[k]
            self.surrogate_distribution = build_surrogate_posterior(
                self.prior_distribution, bijectors=bijectors, initializers=initializers
            )
            return

        def index_persons(self, param, person):
             shape  =  param.shape.as_list()
              trans  =  [1, 0] + list(range(2, len(shape)))
            param  =  tf.transpose(param, trans)
            param  =  tf.gather(param, person)
            param  =  tf.transpose(param, trans)

            return param

        def predictive_distribution(self, data, **params):
            if "_preprocessed" not in data.keys():
                data = self.preprocess(data)
            mu0_gaussian = params["mu0_gaussian"]
            mu00_gaussian = params["mu00_gaussian"]
            beta_gaussian = params["beta_gaussian"]
            beta_poisson = params["beta_poisson"]
            beta_outcome = params["beta_outcome"]
            alpha_outcome = params["alpha_outcome"]

            mu0_poisson = params["mu0_poisson"]
            mu00_poisson = params["mu00_poisson"]

            sd = params["sd00_gaussian"]
            person = data["person_id"]
            # sd = self.index_persons(sd, person)

            mu0_gaussian = self.index_persons(mu0_gaussian, person)
```

```
320
321             mu0_poisson = self.index_persons(mu0_poisson, person)
322
323             mu_gaussian = mu0_gaussian + mu00_gaussian
324             mu_poisson = mu0_poisson + mu00_poisson
325
326             if len(self.gaussian_yearly_vars) > 0:
327                 gaussian_yearly = (
328                     tf.cast(data["gaussian_yearly"], self.dtype)
329                     - self.gaussian_yearly_vars_diff_loc[:, tf.newaxis]
330                 ) / self.gaussian_yearly_vars_scale[:, tf.newaxis]
331                 # plug-in using imputation model
332                 gaussian_yearly_ = gaussian_yearly + tf.zeros_like(mu_gaussian)
333                 gaussian_yearly_ = tf.where(
334                     tf.math.is_finite(gaussian_yearly_), gaussian_yearly_, mu_gaussian
335                 )
336             else:
337                 gaussian_yearly_ = 0
338             if len(self.poisson_yearly_vars) > 0:
339                 poisson_yearly = data["poisson_yearly"]
340                 poisson_yearly_ = poisson_yearly + tf.zeros_like(mu_poisson)
341                 poisson_yearly_ = tf.where(
342                     tf.math.is_finite(poisson_yearly_),
343                     poisson_yearly_,
344                     tf.math.exp(mu_poisson),
345                 )
346             else:
347                 poisson_yearly_ = 0
348
349             if len(self.baseline_vars) > 0:
350                 baseline = tf.cast(data["baseline_vars"], self.dtype)
351                 baseline = baseline[tf.newaxis, ..., tf.newaxis]
352             else:
353                 baseline = 0
354
355             if len(self.binary_yearly_vars) > 0:
356                 has_yearly = tf.cast(data["binary_yearly"], self.dtype)
357             else:
358                 has_yearly = 0
359
360             # regressors
361             ## gaussian yearly predictors
362
363             gaussian_ea = tf.reduce_sum(
364                 beta_gaussian[:, tf.newaxis, : len(self.baseline_vars), :] * baseline,
365                 axis=-2,
366             )[
367                 ..., tf.newaxis
368             ] # batch x person x n_gaussian x year (1)
369             if len(self.binary_yearly_vars) > 0:
370                 gaussian_has = tf.reduce_sum(
371                     beta_gaussian[
372                         :,
373                         tf.newaxis,
374                         len(self.baseline_vars) : len(
375                             self.baseline_vars + self.binary_yearly_vars
```

```
                        ),
                    ][..., tf.newaxis]
                    * has_yearly[..., tf.newaxis, :],
                    axis=-3,
                )
                poisson_has = tf.reduce_sum(
                    beta_poisson[
                        :,
                        tf.newaxis,
                        len(self.baseline_vars) : len(
                            self.baseline_vars + self.binary_yearly_vars
                        ),
                    ][..., tf.newaxis]
                    * has_yearly[..., tf.newaxis, :],
                    axis=-3,
                )
                outcome_has = tf.reduce_sum(
                    beta_outcome[
                        ...,
                        tf.newaxis,
                        len(self.baseline_vars) : len(
                            self.baseline_vars + self.binary_yearly_vars
                        ),
                        tf.newaxis,
                    ]
                    * has_yearly[..., 1:],
                    axis=-2,
                )
            else:
                gaussian_has = 0
                poisson_has = 0
                outcome_has = 0

            if len(self.poisson_yearly_vars) > 0:
                gaussian_poisson = tf.reduce_sum(
                    beta_gaussian[
                        :, tf.newaxis, len(self.baseline_vars + self.binary_yearly_vars) :
                    ][..., tf.newaxis]
                    * (
                        poisson_yearly_[..., tf.newaxis, :]
                        - self.poisson_yearly_vars_loc[:, tf.newaxis, tf.newaxis]
                    )
                    / self.poisson_yearly_vars_scale[:, tf.newaxis, tf.newaxis],
                    axis=-3,
                )
                outcome_poisson = tf.reduce_sum(
                    beta_outcome[
                        ...,
                        tf.newaxis,
                        len(self.baseline_vars + self.binary_yearly_vars) : len(
                            self.baseline_vars
                            + self.binary_yearly_vars
                            + self.poisson_yearly_vars
                        ),
```

```
                        tf.newaxis,
                    ]
                    * (
                        poisson_yearly_[..., 0:1]
                        - self.poisson_yearly_vars_loc[:, tf.newaxis]
                    )
                    / self.poisson_yearly_vars_scale[:, tf.newaxis],
                    axis=-2,
                )

                delta_poisson_yearly = poisson_yearly_[..., 1:] - poisson_yearly_[..., 0:1]
                outcome_poisson_delta = tf.reduce_sum(
                    beta_outcome[
                        ...,
                        tf.newaxis,
                        len(
                            self.baseline_vars
                            + self.binary_yearly_vars
                            + self.poisson_yearly_vars
                        ) : len(
                            self.baseline_vars
                            + self.binary_yearly_vars
                            + self.poisson_yearly_vars
                            + self.poisson_yearly_vars
                        ),
                        tf.newaxis,
                    ]
                    * (delta_poisson_yearly - self.poisson_yearly_vars_loc[:, tf.newaxis])
                    / self.poisson_yearly_vars_scale[:, tf.newaxis],
                    axis=-2,
                )

            else:
                gaussian_poisson = 0
                outcome_poisson = 0
                outcome_poisson_delta = 0

            mu_gaussian += gaussian_ea + gaussian_has + gaussian_poisson

            ## poisson yearly predictors

            poisson_ea = tf.reduce_sum(
                beta_poisson[:, tf.newaxis, : len(self.baseline_vars), :] * baseline,
                axis=-2,
            )[
                ..., tf.newaxis
            ] # batch x person x n_gaussian x year (1)

            if len(self.gaussian_yearly_vars) > 0:
                poisson_gaussian = tf.reduce_sum(
                    beta_poisson[
                        :, tf.newaxis, len(self.baseline_vars + self.binary_yearly_vars) :
                    ][..., tf.newaxis]
                    * (
                        gaussian_yearly_[..., tf.newaxis, :]-self.gaussian_yearly_vars_loc[:, tf.newaxis, tf.newaxis]
```

```
                )
                / self.gaussian_yearly_vars_scale[:, tf.newaxis, tf.newaxis],
                axis=-3,
            )
            outcome_gaussian = tf.reduce_sum(
                beta_outcome[
                    ...,
                    tf.newaxis,
                    len(
                        self.baseline_vars
                        + self.binary_yearly_vars
                        + self.poisson_yearly_vars
                        + self.poisson_yearly_vars
                      ) :  len(
                        self.baseline_vars
                        + self.binary_yearly_vars
                        + self.poisson_yearly_vars
                        + self.poisson_yearly_vars
                        + self.gaussian_yearly_vars
                       ),
                    tf.newaxis,
                ]
                * (
                    gaussian_yearly_[..., 0:1]
                    - self.gaussian_yearly_vars_loc[:, tf.newaxis]
                )
                / self.gaussian_yearly_vars_scale[:, tf.newaxis],
                axis=-2,
            )
            delta_gaussian_yearly = (
                gaussian_yearly_[..., 1:] - gaussian_yearly_[..., 0:1]
            )
            outcome_gaussian_delta = tf.reduce_sum(
                beta_outcome[
                    ...,
                    tf.newaxis,
                    len(
                        self.baseline_vars
                        + self.binary_yearly_vars
                        + self.poisson_yearly_vars
                        + self.poisson_yearly_vars
                        + self.gaussian_yearly_vars
                      ) :,
```

```
                    tf.newaxis,
                ]
                * (
                    delta_gaussian_yearly
                    - self.gaussian_yearly_vars_diff_loc[:, tf.newaxis]
```

```
                    )
                    / self.gaussian_yearly_vars_diff_scale[:, tf.newaxis],
                    axis=-2,
                )
            else:
                poisson_gaussian = 0
                outcome_gaussian = 0
                outcome_gaussian_delta = 0

            mu_poisson += (
                poisson_ea
                + poisson_has
                + poisson_gaussian
                - tf.math.log(self.poisson_yearly_vars_scale)[..., tf.newaxis]
            )

            ## outcome
            outcome_ea = tf.reduce_sum(
                beta_outcome[..., tf.newaxis, : len(self.baseline_vars), tf.newaxis]
                * baseline,
                axis=-2,
            )

            # model the values
            if len(self.gaussian_yearly_vars) > 0:
                rv_gaussian = tfd.Normal(mu_gaussian, sd)
                ll_gaussian = rv_gaussian.log_prob(gaussian_yearly)
                ll_gaussian = tf.where(
                    tf.math.is_finite(ll_gaussian), ll_gaussian, tf.zeros_like(ll_gaussian)
                )
                # ll_gaussian *= tf.cast(tf.math.is_finite(gaussian_yearly), self.dtype)
                ll_gaussian = tf.reduce_mean(ll_gaussian, axis=[-1, -2])
            else:
                ll_gaussian = 0
            if len(self.poisson_yearly_vars) > 0:
                rv_poisson = tfd.Poisson(log_rate=mu_poisson)
                ll_poisson = rv_poisson.log_prob(poisson_yearly)
                ll_poisson = tf.where(
                    tf.math.is_finite(ll_poisson), ll_poisson, tf.zeros_like(ll_poisson)
                )

                ll_poisson  *= tf.cast(tf.math.is_finite(poisson_yearly), self.dtype)
                ll_poisson  = tf.reduce_mean(ll_poisson, axis=[-1, -2])
             else:

                ll_poisson  = 0

            # model the outcome
            mu_outcome = (
                outcome_gaussian
                + outcome_ea
                + outcome_poisson
                + outcome_has
                + alpha_outcome[..., tf.newaxis]
                + outcome_gaussian_delta
                + outcome_poisson_delta
            )
```

```
            outcome = data[self.outcome_var][..., 1:]
            mu_outcome += tf.zeros_like(outcome)
            rv_outcome = tfd.Bernoulli(logits=mu_outcome)
            ll_outcome = rv_outcome.log_prob(outcome)
            ll = ll_gaussian + ll_poisson + tf.reduce_sum(ll_outcome, axis=-1)
            return {
                "log_likelihood": ll,
                "ll_outcome": ll_outcome,
                "ll_poisson": ll_poisson,
                "ll_gaussian": ll_gaussian,
                "rv_outcome": rv_outcome,
            }

    def log_likelihood(self, data, **params):
            return self.predictive_distribution(data, **params)["log_likelihood"]

```

```
    def unormalized_log_prob(self, data=None, prior_weight=tf.constant(1.0), **params):
            prediction = self.predictive_distribution(data, **params)
            log_likelihood = prediction["log_likelihood"]
            max_val = tf.reduce_max(log_likelihood)

            finite_portion = tf.where(
                tf.math.is_finite(log_likelihood),
                log_likelihood,
                tf.zeros_like(log_likelihood),
            )
            min_val = tf.reduce_min(finite_portion) - 1.0
            log_likelihood = tf.where(
                tf.math.is_finite(log_likelihood),
                log_likelihood,
                tf.ones_like(log_likelihood) * min_val,
            )
            prior = self.prior_distribution.log_prob(params)
            prior_weight = tf.cast(prior_weight, self.dtype)
            return tf.reduce_sum(log_likelihood, axis=-1) + prior_weight * prior

    def reverse_kl(self, data, model, **params):
            prediction = self.predictive_distribution(data, **params)["rv_outcome"]
            other_prediction = model.predictive_distribution(data, **params)["rv_outcome"]
            kl = other_prediction.kl_divergence(prediction)
            return kl

    def fit_projection(
            self, other, batched_data_factory, num_steps, samples=32, **kwargs
    ):
            def objective(data):
                this_prediction = self.predictive_distribution(
                    data, **self.sample(samples)
                )["rv_outcome"]
                other_prediction = other.predictive_distribution(
                    data, **other.sample(samples)
                )["rv_outcome"]
                delta = other_prediction.kl_divergence(this_prediction)
                return tf.reduce_mean(delta)
```

```
        return batched_minimize(
            objective,
            batched_data_factory=batched_data_factory,
            num_steps=num_steps,
            trainable_variables=self.surrogate_distribution.variables,
            **kwargs,
        )

    def preprocess(self, record):
        out = {
            self.outcome_var: record[self.outcome_var],
            "person_id": record["person_id"],
        }
        if len(self.gaussian_yearly_vars) > 0:
            out["gaussian_yearly"] = tf.concat(
                [
                    tf.cast(record[c][..., tf.newaxis, :], self.dtype)
                    for c in self.gaussian_yearly_vars
                ],
                axis=-2,
            )
        if len(self.poisson_yearly_vars) > 0:
            out["poisson_yearly"] = tf.concat(
                [
                    tf.cast(record[c][:, tf.newaxis], self.dtype)
                    for c in self.poisson_yearly_vars
                ],
                axis=-2,
            )
            if len(self.baseline_vars)  > 0:
                out["baseline_vars"] =  tf.concat(
                [
                    tf.cast(record[c][:, tf.newaxis], self.dtype)
                    for c in self.baseline_vars
                ],
                axis=-1,
            )
```

```
        if len(self.binary_yearly_vars) > 0:
            out["binary_yearly"] = tf.concat(
                [
                    tf.cast(record[c][:, tf.newaxis], self.dtype)
                    for c in self.binary_yearly_vars
                ],
                axis=-2,
            )
        return out

    def loo_auc(self, data_factory, samples=32):
        ll = []
        labels = []
        predicted = []
```

```
            _p = self.sample(samples)
            for batch in tqdm(iter(data_factory())):
                pred = self.predictive_distribution(batch, **_p)
                ll += [pred["ll_outcome"]]
                predicted += [pred["rv_outcome"].prob(1.0)]
                labels += [batch[self.outcome_var][:, 1:]]
            ll = tf.concat(ll, axis=1)
            predicted = tf.concat(predicted, axis=1)

            labels = tf.concat(labels, axis=0)
            finite_portion = tf.where(
                tf.math.is_finite(ll),
                ll,
                tf.zeros_like(ll),
            )
            min_val = tf.reduce_min(finite_portion) - 1.0
            ll = tf.where(
                tf.math.is_finite(ll),
                ll,
                tf.ones_like(ll) * min_val,
            )

            lw, khat = psislw(-tf.reduce_sum(ll, axis=-1).numpy())
            w = tf.math.exp(lw)
            w = (w / tf.reduce_sum(w, axis=0, keepdims=True))[..., tf.newaxis]

            psis_pred_y = tf.reduce_sum(predicted * w, axis=0)
            loo = psisloo(tf.reduce_sum(ll, axis=-1).numpy())
            print(f"loo: {loo}")

            predicted = tf.reduce_mean(predicted, axis=0)
            predicted = np.reshape(predicted, -1)
            psis_pred_y = np.reshape(psis_pred_y, -1)
            labels = np.reshape(labels, -1)
            loo_roc = auroc(labels, psis_pred_y)
            loo_prc = auprc(labels, psis_pred_y)

            roc = auroc(labels, predicted)
            prc = auprc(labels, predicted)

            return {
                "loo": loo,
                "loo_roc": loo_roc,
                "loo_prc": loo_prc,
                "prc": prc,
                "roc": roc,
                "khat": khat,
            }


def auroc(labels, probs):
    fpr, tpr, thresholds = metrics.roc_curve(labels, probs, pos_label=1)
    return {
        "auroc": metrics.auc(fpr, tpr),
        "fpr": fpr,
        "tpr": tpr,
        "thresholds": thresholds,
```

```
    }


def auprc(labels, probs):
    precision, recall, thresholds = metrics.precision_recall_curve(labels, probs)
    return {
        "auprc": metrics.auc(recall, precision),
        "precision": precision,
        "recall": recall,
        "thresholds": thresholds,
    }


def classification_metrics(
    data_factory,
    prediction_fn,
    by_vars=None,
    outcome_label="label",
    save_file=None,
):
    if by_vars is None:
        by_vars = []
    collect_vars = by_vars + [outcome_label]
    collect_vars = set(collect_vars)
    collected_data = {k: [] for k in collect_vars}
    probs = []
    metrics = {}

    for batch in iter(data_factory()):
        for k in collected_data.keys():
            collected_data[k] += [tf.squeeze(batch[k]).numpy()]

        probs += [prediction_fn(data=batch)]

    probs = np.concatenate(probs, axis=0)
    for k in collected_data.keys():
        collected_data[k] = np.squeeze(np.concatenate(collected_data[k], axis=0))

    # HACK
    probs = tf.reshape(probs, -1)
    collected_data[outcome_label] = tf.reshape(collected_data[outcome_label][:, 1:], -1)

    computed = pd.DataFrame({"probs": probs, **collected_data})
    if save_file:
        computed.to_parquet(save_file)
    metrics["prob"] = np.mean(computed[outcome_label])
    metrics["auroc"] = auroc(computed[outcome_label], computed.probs)
    metrics["auprc"] = auprc(computed[outcome_label], computed.probs)
    return metrics


```

References

[1] Piironen J, Vehtari A. On the Hyperprior Choice for the Global Shrinkage Parameter in the Horseshoe Prior. arXiv:161005559 [stat]. 2016 Oct;.

[2] Piironen J, Vehtari A. Sparsity Information and Regularization in the Horseshoe and Other Shrinkage Priors. Electronic Journal of Statistics. 2017;11(2):5018–5051.

[3] Catalina A, Bu¨rkner PC, Vehtari A. Projection Predictive Inference for Generalized Linear and Additive Multilevel Models. In: Proceedings of The 25th International Conference on Artificial Intelligence and Statistics. PMLR; 2022. p. 4446–4461.

[4] Pavone F, Piironen J, Bu¨rkner PC, Vehtari A. Using Reference Models in Variable Selection. Computational Statistics. 2023 Mar;38(1):349–371.

[5] Piironen J, Paasiniemi M, Vehtari A. Projective Inference in High-Dimensional Problems: Prediction and Feature Selection. Electronic Journal of Statistics. 2020 Jan;14(1):2155–2197.

[6] Piironen J, Vehtari A. Comparison of Bayesian Predictive Methods for Model Selection. Statistics and Computing. 2017 May;27(3):711–735.

[7] Weber F, Glass A, Vehtari A. Projection Predictive Variable Selection for Discrete Response Families¨ with Finite Support. Computational Statistics. 2025 Feb;40(2):701–721.
