# Supplementary material for: Functional improvement is a better predictor of steady work than medical improvement for individuals with mental health conditions
Source: PLOS Ment Health. 2025 Aug 5;2(8):e0000384. doi: 10.1371/journal.pmen.0000384 (PMC12798178; doi:10.1371/journal.pmen.0000384)
Supplement: Attachment — Submitted filename: response letter.pdf [file pmen.0000384.s003.pdf]

PMEN-D-25-00182
Title: Functional improvement is a better predictor of steady work than medical improvement for individuals with mental health conditions

We would like to thank the editor and reviewers for their efforts in reviewing our manuscript. In addressing the reviewer comments, we have made improvements in clarity of exposition which we believe will help readers of this journal better-understand the results and implications of our manuscript. Please see below for detailed responses to the reviews:

Response to Reviewer 1

Specific comments.

1. *First, while the statistical methods and results are presented in a highly technical manner, the methods are so rigorous that they may not be easily accessible to the general readership of PLOS Mental Health. Consider simplifying explanations, summarizing key methodological decisions in the main text (e.g., the imputation strategy, feature scaling, rationale for variable selection), and referring to supplemental material only for technical details.*

   Response. We have simplified the explanations for the technical terms. We have also summarized the imputation, feature scaling, and rationale for selecting the variables which are influential.

   We incorporated the new text:

   > **On scaling (line 95):** To facilitate comparisons of effect sizes, we scaled baseline values of predictors by subtracting the mean and dividing by the standard deviation within our models.
   > **On selection (line 100):** To facilitate comparisons of effect sizes, we scaled baseline values of predictors by subtracting the mean and dividing by the standard deviation within our models.
   > **On imputation (line 108):** As a result of scaling, we are easily able to identify the impactful variables in our models by ranking the magnitude of their corresponding regression coefficients.
   > **On cross validation (line 135):** We adjudicated the models on predictive accuracy using Bayesian leave one out (LOO) cross validation (CV). LOO-CV is a method used to evaluate models by training them multiple times, with one data point left out from the dataset during each training iteration. We estimated the LOO prediction for each observation modeled (29,30).

2. *Similarly, expanding the interpretation of the neural network results and their added value over logistic regression could enhance understanding.*

Response. We have explained the difference between neural network and logistic regression, and the added value of the neural network.

> **Line 122:** To ensure that our results are not due to the limitations of linear regression, we also replicated the same analysis using two nonlinear modeling techniques to investigate the consistency of results across different models. First, we fit Bayesian artificial neural networks to the same data with a hidden layer size of 12. Second, we fit a piecewise Bayesian generalized linear model based on an additive method (27,28) that captures nonlinear effects in model coefficients. The model fits a separate regression model to each local region of the data. The difference between these three models lies in the assumptions of the models. The logistic regression assumes a linear relationship between predictors and the log-odds of the outcome. The piecewise logistic regression and neural networks do not assume linearity in this relationship and provide flexible frameworks capable of capturing complex, non-linear associations and higher-order interactions among covariates.

3. *Second, while the Discussion section is rich in implications, it could benefit from more critical reflection on why medical predictors underperformed. Is this due to measurement limitations, a conceptual mismatch, or perhaps the use of raw variables instead of derived composites? Clarifying this would strengthen the practical relevance of your findings.*

   Response. We have added language to the discussion section to clarify the value of the functional profile measure generated by the WD-FAB as compared to medical condition variables. WD-FAB measures how someone's physical and mental health limitations affect their ability to work. The results imply that by adjusting for the subdomains of WD-FAB, medical conditions are no longer significantly related to the outcome.

   > **Line 332:** The WD-FAB characterizes how someone's physical and mental health limitations affect their ability to work along eight key subdomains of function: Basic Mobility, Upper Body Function, Fine Motor Function, Communication & Cognition, Resilience & Sociability, Self-Regulation, and Mood & Emotions. The results imply that by adjusting for the subdomains of the WD-FAB, which reflect an overall functional profile, medical conditions are no longer strongly related to the outcome.

4. *Additionally, results such as the positive predictive value of BMI warrant further exploration to avoid misinterpretation by readers. You may also wish to expand on how the WD-FAB might beented in real-world SSA evaluations or workplace settings.*

   Response. Unlike work-related physical and mental function, the nature of the relationship between BMI and ability to work is unclear. For instance, for some job types having a high BMI might be an impediment to being able to work. However, certain debilitating physical conditions such as cancer or metabolic disorders may cause one to have a low BMI, where these disorders also impair physical/mental function in a way that makes work infeasible. In our results, we found that a positive change in BMI has a positive predictive value in determining steady work for an individual. Notably, the BMI effect is muted in the model incorporating all variables relative to the model consisting of demographic + medical variables. This finding suggests that the increase in BMI is often reflected in changes in functional measures.

We have incorporated the following text into our Discussion:

> **Line 313:** Unlike work-related physical and mental function, the nature of the relationship between BMI and ability to work is unclear. For instance, for some job types, having a high BMI might be an impediment to being able to work. While high BMI is generally associated with lower physical function (33), this relationship is not monotic (34). Certain debilitating physical conditions such as cancer or metabolic disorders may cause one to have a low BMI, where these disorders also impair physical/mental function in a way that makes work infeasible. In fact, there is evidence that having a higher BMI is can be protective with respect to mortality (35,36). Notably, when functional measures are removed (Fig 5), then the change in BMI becomes a much stronger predictor going from an average odds ratio of approximately 1.2 to approximately 2.1. This finding suggests that the increase (or decrease) of BMI is often associated to changes in the functional measures.
>
> 33. Zhang X, Brown JC, Schmitz KH. Association between Body Mass Index and Physical Function among Endometrial Cancer Survivors. PLOS ONE. 2016 Aug 16;11(8):e0160954.
> 34. Kıskaç M, Soysal P, Smith L, Capar E, Zorlu M. What is the Optimal Body Mass Index Range for Older Adults? Ann Geriatr Med Res. 2022 Mar;26(1):49–57.
> 35. Elbaz-Greener G, Rozen G, Carasso S, Yarkoni M, Wijeysundera HC, Alcalai R, et al. The Relationship Between Body Mass Index and In-hospital Survival in Patients Admitted With Acute Heart Failure. Front Cardiovasc Med. 2022 Apr 28;9:855525.

5. *Finally, the manuscript is well written overall, but the clarity and narrative flow, feasibly implemparticularly in dense sections, would benefit from some restructuring and simplification. Figures should be explained more intuitively, and technical terms such as "marginalizing over missing values" or "Pareto-smoothed importance sampling" could be briefly defined or paraphrased for clarity.*

   <u>Response.</u> We have paraphrased technical terms and in certain cases removed them entirely from the main text.

## Response to Reviewer 2

We appreciate very much your suggestions and comments that we believe have improved our manuscript significantly. Our point-to-point responses to you are below.

1. *This manuscript meets the scientific, ethical, and reporting standards for publication in PLOS Mental Health however a light language edit focusing on sentence flow and stylistic consistency is needed.*

Response. We have performed a round of copy-editing in this revision. We hope that it will be to your satisfaction.
