# Supplementary material for: Functional improvement is a better predictor of steady work than medical improvement for individuals with mental health conditions
Source: PLOS Ment Health. 2025 Aug 5;2(8):e0000384. doi: 10.1371/journal.pmen.0000384 (PMC12798178; doi:10.1371/journal.pmen.0000384)
Supplement: Attachment — Submitted filename: response letter-r2.pdf [file pmen.0000384.s004.pdf]

PMEN-D-25-00182
Title: Functional improvement is a better predictor of steady work than medical improvement for individuals with mental health conditions

Response to Reviewer 1

Specific comments.

1. *First, in the introduction, the authors may consider adding one brief sentence to more explicitly highlight that direct comparisons between functional and medical improvements as predictors of employment are limited in the current literature — this would better frame the unique contribution of the study.*

   Response. We have added a sentence at the end of the introduction (line 71): "Our goal was to understand the predictive power of functional improvement versus medical improvement on work outcomes with a particular focus on steady work (12–19) since, historically, the role of functional improvement is rarely considered, especially in relationship to medical improvement."

2. *Second, while the methods are clearly described, a very brief statement explaining the rationale for using Bayesian methods (e.g., their flexibility with complex data and missing values) may help readers less familiar with these approaches. Similarly, a short sentence clarifying why the specific nonlinear models (piecewise regression and Bayesian neural networks) were selected would be helpful for completeness.*

   Response. We have added a brief statement at the beginning of Statistical analyses section (line 88): "Bayesian methods handle missing values of predictors by averaging over estimates of these values. They are also able to use sparsity in order to find parsimonious models that are easier to understand."

   In addition, we added a sentence to clarify the selection on the methods in the Beyond linear regression section (line 126): "The reason for selecting these two nonlinear methods is that they both are piecewise linear models. For this reason they are related-to but also extend the generalized linear model in relaxing the assumptions between relationships."

3. *Third, although the discussion appropriately acknowledges the complex role of BMI, adding one or two lines further acknowledging possible reverse causality (e.g., weight changes as a result of functional decline) would strengthen the interpretation of these findings.*

   Response. We have added a sentence in Discussion (line 266): "Potentially, BMI and function are inter-related causal factors impacting the ability to work. For instance, functional decline could lead to weight gain which could then impact work ability."

4. *Lastly, the policy implications are well addressed; however, a brief addition about how these findings could inform disability determination processes in practice would increase the applied relevance.*

Response. We have added a sentence at the end of the conclusion (line 304): “In addition, determining one’s ability to work is an important step in the disability determination process. The identified factors that significantly impact the ability to work will provide valuable insights in the determination.”
